# Patients with Papillary Renal Cancer and Germline Duplication of *MET* Exons 5-21

**DOI:** 10.3390/biomedicines13061329

**Published:** 2025-05-29

**Authors:** Dmitry S. Mikhaylenko, Natalya B. Kuryakova, Fatima M. Bostanova, Viktoria V. Zabnenkova, Oksana P. Ryzhkova, Ilya V. Volodin, Dmitry V. Zaletaev, Dmitry V. Pustoshilov, Sergey I. Kutsev, Vladimir V. Strelnikov

**Affiliations:** 1 Research Centre for Medical Genetics, Moskvorechie st., 1, 115522 Moscow, Russiavstrel@list.ru (V.V.S.); 2 Department of Translational Medicine and Biotechnology, Sechenov University, Trubetskaya St., 119991 Moscow, Russia; 3 “Biotech Campus” LLC., Mikluho-Maklaya st., 117437 Moscow, Russia

**Keywords:** hereditary papillary renal cancer, gene *MET*, germline duplication, next generation sequencing

## Abstract

Hereditary papillary renal carcinoma (HPRC) is a rare monogenic hereditary disease in the group of hereditary cancer syndromes. Clinically, HPRC results in the development of multiple papillary renal cell carcinomas of the kidneys in young adults. HPRC is caused by point activating mutations in the *MET* gene encoding a transmembrane tyrosine kinase receptor. Until now, all detected germline mutations in HPRC patients were missense variants leading to a constitutive activation of the tyrosine kinase domain. We describe, for the first time, unrelated patients with clinical features similar to HPRC and without *MET* pathogenic missense variants but harboring an extended heterozygous duplication ~101.4 kb in length (chr7:116740252-116841718) in 7q31.2 determined using whole-genome sequencing (WGS). This duplication results in an additional copy of the *MET* gene fragment, including exons 5-21. The duplicated exons encode most of the receptor domains. According to the American College of Medical Genetics and Genomics (ACMG) criteria, this duplication is classified as variant of uncertain significance (VUS) at present, but it is not excluded that this duplication may represent an activating mutation. Perhaps, further segregation analysis and functional studies will allow us to more accurately resolve the pathogenicity and diagnostic significance of this germline CNV.

## 1. Introduction

More than 90 thousand cases of renal cancer (RC) and about 33 thousand deaths from this disease were registered in the European Union in 2022, which allows us to consider RC to be one of the main problems in modern oncologic urology [1]. About 3% of cases of RC are manifestations of a hereditary cancer syndrome associated with tumors at a young age and/or multiple kidney tumors. Among the most well-known hereditary cancer syndromes with a high risk of developing RC and an autosomal dominant type of inheritance are von Hippel–Lindau syndrome, Birt–Hogg–Dubé syndrome, hereditary papillary renal cell carcinoma (HPRC), hereditary syndrome with fumarate hydratase deficiency (previously called hereditary leiomyomatosis and renal cell carcinoma), and some others [2]. HPRC is not the most common form of hereditary RC, but in the vast majority of HPRC cases it is associated with the development of primary multiple tumors in both kidneys. A systematic review of publications on primary multiple RC in 7689 patients showed that multiple papillary carcinomas occur in 16.1% of cases, which significantly exceeds the expected frequency calculated based on the representation of various types of RC in the general population [3]. We have also previously described a case of HPRC in a Russian patient [4].

HPRC is caused by a germline-activating mutation in the *MET* oncogene, localized in the 7q31 region. *MET* encodes a transmembrane tyrosine kinase receptor that is involved in signaling pathway resulting in the stimulation of cell division and in the repair of the renal tubular epithelium after ischemic or chemical damage. The *MET* gene contains 21 exons. The protein coding sequence begins in exon 2. Briefly, exons 2-5 encode a semaphorin homology (SEMA) domain, exons 6–12 encode an immunoglobulin-like domain, exon 13 a trans-membrane domain, exon 14 a juxtamembrane domain, exons 15-20 encode a tyrosine kinase domain, and exon 21 a multifunctional docking site. The ligand for MET is hepatocyte growth factor, which binds to the SEMA domain, causing the homodimerization and activation of the receptor tyrosine kinase domain [5]. The currently described germline *MET* pathogenic (P)/likely pathogenic (LP) mutations causative for HPRC are heterozygous missense variants in exons 15-21, which lead to the dimerization, autophos-phorylation, and constitutive activation of the receptor tyrosine kinase domain (V1092I, H1112R, M1149T, V1206L, V1238I, D1246N, Y1248C, M1268T, and others; OMIM *164860). Activated MET triggers signaling through the PI3K/AKT/mTOR, STAT, MEK/ERK, GRB2, GAB1, and RAC1 pathways, which promotes cell proliferation, motility, and angiogenesis [6].

Similar somatic *MET* missense mutations are detected in sporadic papillary renal carcinomas in no more than 13% of cases, although, amplification, point mutations, and/or overexpression of the *MET* gene are detected in more than 80% of sporadic papillary renal carcinomas, indicating an important role of MET activation in the carcinogenesis of these tumors [2,6,7,8]. However, we were unable to find any published data about germline *MET* copy number abnormalities that would cause HPRC. Here, we present the first description of a germline duplication of a large part of the *MET* gene identified by using whole-genome sequencing (WGS) in patients with clinical features of HPRC.

## 2. Materials and Methods

### 2.1. Anamnesis

We examined two patients from unrelated Russian families who underwent medical genetic counseling and molecular genetic testing.

**Figure 1:**
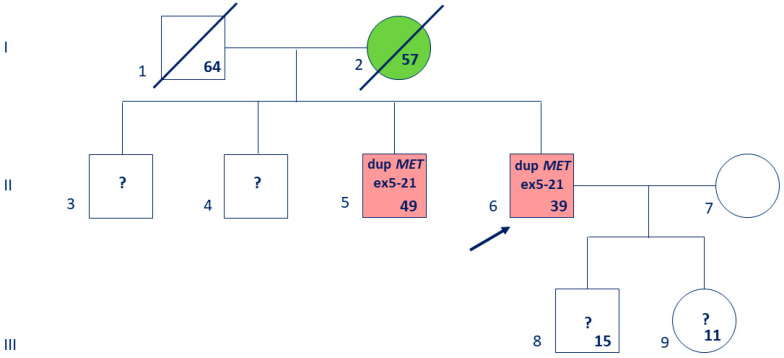
Pedigree of a proband with suspected HPRC; Case #1. Legend: the proband is marked by an arrow; deceased family members are crossed out, with the age indicated; the age on the date of counseling is signed at the bottom right; generations are numbered in Roman numerals; individuals are numbered in Arabic numerals; endometrial cancer is shown in green; multiple papillary RC is shown in red; “?”—MET duplication status is unknown.

**Case #1.** The patient was a 39-year-old man. Renal parenchyma neoplasms were detected in the patient at the age of 37, in 2022: 5 tumors ranging in size from 8 to 50 mm in the right kidney and 8 tumors from 4 to 44 mm in the left kidney. The patient was tested for *VHL* point mutations in another laboratory and germline mutations were not detected. Biopsy of one of the tumors from the right kidney was performed in 2023; the results of the pathological examination indicated papillary renal cell carcinoma, Fuhrman G1. Then, the patient underwent laparoscopic multiple resection of the right kidney, and all detected tumors were removed. According to the pathological examination, these neoplasms were multiple papillary renal cell carcinomas, Grade 1, according to WHO/ISUP. The largest tumor size was 6.5 cm, without signs of vascular invasion. Rehospitalization was recommended for resection of the tumors in the left kidney. The patient has a positive family history of cancer. He has a 49-year-old brother diagnosed with multiple papillary carcinomas in both kidneys (T2N0M0) who was previously tested for point mutations in the *MET* and *FH* genes by Sanger sequencing in our laboratory, and P/LP germline variants were not identified. The patient’s mother died at the age of 57 due to the progression of endometrial carcinoma (Figure 1). According to the data obtained from the patient, other relatives shown in the pedigree do not have cancer. Thus, the pedigree established by the clinical and genealogical analysis indicates a possible diagnosis of HPRC.

**Case #2.** The patient was a 36-year-old man. At the age of 34, he was diagnosed with cysts in both kidneys, angiomyolipoma in the left kidney, and a neoplasm in the upper pole of the right kidney according to ultrasound and MRI data. Laparoscopic resection of his right kidney with 5 tumors was performed after 2 years. According to the pathological examination, all removed tumors were papillary renal carcinoma, Fuhrman G2. He has a hyperpigmented spot 1 × 3 cm in the lumbar region. Familial cancer history is absent. Previously, the patient underwent sequencing and MLPA of the *VHL* and *TSC1/2* genes at another laboratory, with a negative result. The indications for excluding von Hippel–Lindau syndrome and tuberous sclerosis were polycystic kidney disease and angiomyolipoma at a relatively young age.

### 2.2. Genetic Laboratory Testing

**Isolation of genomic DNA:** Genomic DNA was isolated from the blood using the Gen-taMag Blood HMW DNA kit (“Genterra”, Moscow, Russia) by sorption on magnetic particles. The concentration of the obtained DNA samples was measured by the fluorometric method on a Qubit 3.0 fluorometer (“ThermoFisherScientific”, Waltham, MA, USA).

**Analysis of mutations in the *MET* exons 15-21 and *FH* genes:** Polymerase chain re-action (PCR) was performed in a C1000 thermal cycler (“BioRad”, Hercules, CA, USA). The reagents were 2.5 μL 10× buffer for PCR (“SibEnzyme”, Novosibirsk, Russia), 1.5 μL 25 mM MgCl_2_, 2 μL dNTPs (mix containing 2.5 mM each), 5 pmol forward and reverse primers, 2.5 a.u. Taq-polymerase, 1 μL genomic DNA, and up to 25 μL deionized water. The primer sequences for amplification of the *MET* exons 15-21 and exons 1-10 of the *FH* gene were identical to those used previously [4,9]. PCR temperature parameters were as follows: initial denaturation at 95 °C for 2.5 min, then 38 cycles (95 °C, 45 s; 60 °C, 30 s; 72 °C, 30 s), and final elongation at 72 °C for 2 min. PCR products were treated with *E. coli* exonuclease I (2 U; “Fermentas”, Vilnius, Lithuania) and alkaline phosphatase (1 U; “SibEnzyme”, Novosibirsk, Russia) to remove unreacted primers and dNTPs. An aliquot of 2.5 μL of the treated PCR product was used as a template in the Sanger sequencing reaction using the BigDye^®^ Terminator v3.1 Cycle Sequencing Kit (“ThermoFisherScientific”, Waltham, MA, USA) according to the manufacturer’s instructions. Detection of labeled fragments was performed on a 3500 capillary genetic analyzer (“ThermoFisherScientific”, Waltham, MA, USA). Sanger sequencing chromatograms were analyzed using the Chromas v2.6.6 software (“Technelysium”, Brisbane, Australia), and the sequences were aligned against the reference sequence of *MET* (NM_000245.4) and *FH* (NM_000143.4) using the Ensembl genome browser (https://www.ensembl.org/, accessed on 3 April 2025).

**Whole-genome sequencing (WGS):** WGS was performed using the DNBSEQ-T7RS platform. Genomic DNA was isolated from the blood-containing plates using a MGIEasy Magnetic Beads Genomic DNA Extraction Kit (“MGI”, Shenzhen, China) according to the manufacturer’s protocol. The extracted DNA was quantified using a Qubit^TM^ dsDNA Quantification Assay Kit (“ThermoFisherScientific”, Waltham, MA, USA). Each gDNA sample (1000 ng) was used to construct a genomic DNA library using the MGIEasy Fast PCR-FREE FS DNA Library Prep Set V2.0 (“MGI”, Shenzhen, China) according to the manufacturer’s instructions. DNA was fragmented by enzymatic fragmentation using magnetic beads. DNA end-repair and adapter ligation were conducted using the MGIEasy UDB PF Adapters-96 Kit (“MGI”, Shenzhen, China). The products were run on a 4200 TapeStation using the Agilent D1000 ScreenTape (“Agilent”, Santa Clara, CA, USA) to assess the size distribution of the libraries. They were also quantified using a Qubi^TM^ dsDNA Quantification Assay Kit. The PCR products were circularized and 75 fmol of ssCirDNA was amplified using rolling-circle amplification to generate DNA nanoball-based libraries, which were loaded onto a DNBSEQ-T7RS sequencing flow cell with a DNBSEQ-T7RS High-throughput Sequencing Kit (“MGI”, Shenzhen, China). The library was run on a DNBSEQ-T7RS platform at paired-end 150 bp reads.

### 2.3. Bioinformatic Analysis

**Preprocessing:** Low-quality read ends and adapters were trimmed using cutadapt v.4.2 (https://github.com/marcelm/cutadapt, accessed on 10 March 2025) with parameters “--trim-n --quality-cutoff 30,30 --error-rate 0.1 --times 99 --minimum-length 0 --pair-filter both --interleaved”. Read pair mapping was performed using BWA-mem v.0.7.17 (https://github.com/lh3/bwa, accessed on 15 March 2025) with parameters “-k 30 -K 100000000 -Y” to the human reference genome GRCh38 and sorted with samtools v.1.16.1 (https://github.com/samtools/samtools, accessed on 10 March 2025). Duplicated reads in alignment were marked using MarkDuplicatesSpark from GATK v.4.3.0.0 (https://github.com/broadinstitute/gatk, accessed on 10 March 2025) with default parameters. The quality of the bases in the reads was checked using FastQC v.0.11.9 (www.bioinformatics.babraham.ac.uk/projects/fastqc/, accessed on 10 March 2025) software. In Case #1, there were 1,393,054 total reads with a read length of 2 × 150 bp and ×32 average coverage, of which only 2.01% of the sequences had a less than 10% coverage; in Case #2, there were 1,607,964 total reads with a read length of 2 × 150 bp and ×36 average coverage, of which only 1.64% of the sequences had a less than 10% coverage.

**Variant calling and annotation:** Variant calling was processed with DeepVariant algorithm under Clara Parabricks v.4.0.0 (https://www.NVIDIA.com/en-us/clara/genomics/, accessed on 10 March 2025) with parameters “--disable-use-window-selector-model --normalize-reads --track-ref-reads --min-mapping-quality 10”. The identified genetic variants were designated in accordance with the HGVS nomenclature v.21.0.0 (https://hgvs-nomenclature.org, accessed on 3 April 2025). The NGS-Data-Genome online service with the FIP registration number 2021662119 developed at the Research Centre for Medical Genetics for internal tasks was used to process the genome sequencing data (https://med-gen.ru/en/press-center/news/otdelom-bioinformatiki-mgntc-razrabotana-programma-ngs-data-genome-dlia-obrabotki-dannykh-ekzomnogo-i-polnogenomnogo/, accessed on 3 April 2025). The population frequencies of these variants were obtained from the 1000 Genomes project and GnomAD v.3.1.2 (http://gnomad.broadinstitute.org/, accessed on 10 March 2025) data. The clinical (diagnostic) significance of the variants was evaluated using the OMIM database (http://omim.org, accessed on 3 April 2025) and open publications. In addition, we searched databases containing significant data about germline mutations in hereditary cancer syndromes: Franklin Genoox (https://franklin.genoox.com/clinical, accessed on 3 April 2025), LOVD (https://www.lovd.nl/, accessed on 3 April 2025), Varsome (https://varsome.com/, accessed on 3 April 2025), and ClinVar (http://www.ncbi.nlm.nih.gov/clinvar/, accessed on 3 April 2025).

**Copy number variation analysis:** The copy number variations (CNVs) were studied using bioinformatic tools: Canvas (https://github.com/Illumina/canvas, accessed on 10 March 2025) and the CNV algorithm of the NGS-Data-Genome online service with default parameters.

**Interpretation of the pathogenicity/oncogenicity of genetic variants:** Interpretation of the pathogenicity of genetic variants was performed according to the ACMG [10] and CanVIG guidelines [11]. The oncogenicity of genetic variants was assessed using the recommendations of ClinGen/CGC/VICC [12]; the clinical significance of sequence variants in cancer was assessed according to the AMP/ASCO/CAP recommendations [13].

**Multiplex Ligation-dependent Probe Amplification (MLPA): ***MET* duplication analysis was performed via MLPA using the SALSA MLPA P308 MET kit (MRC Holland, Amsterdam, The Netherlands), which includes 26 probes in the *MET* and flanking sequences, 4 in *PTEN*, and 4 in *LRRK2*, as well as 14 reference probes for other genomic regions not involved in HPRC. Fragment analysis and CNV evaluation were performed using a 3500 Genetic Analyzer (ThermoFisherScientific, Waltham, MA, USA) and Coffalyser v.04 software (MRC Holland, Amsterdam, The Netherlands); the heterozygous duplication interval was set at 1.30 < FR < 1.65.

## 3. Results

### 3.1. Absence of MET Gene Alteration in Routine Testing

**Case #1:** The molecular genetic testing of the *VHL* mutations previously performed for the patient at another laboratory seems to be insufficiently justified, since a pathologist’s conclusion regarding the type of renal cell carcinoma was not received before testing. Previously, we tested the patient’s older brother, diagnosed with multiple papillary RCC, for germline mutations in *MET* exons 15-21. P/LP variants and variants of uncertain significance (VUSs) were not identified. Since the new WHO classification of kidney tumors presented late 2022 [14] was just beginning to enter into practice, we also performed Sanger sequencing of *FH* exons 1-10 for the patient’s brother (*FH* loss-of-function germline mutations cause FH-deficient RC, which was previously called papillary RC type 2). The result of this *FH* point mutation testing was also negative.

**Case #2:** The patient underwent sequencing of exons 15-21 of the *MET* gene. P/LP variants were not detected. Having considered the negative results of routine genetic testing, we proceeded with WGS of the probands’ DNA in Cases #1 and #2. WGS was performed within a research project for which the inclusion criteria were young age and multiple tumors.

### 3.2. Whole-Genome Sequencing Reveals a Large MET Duplication

**Figure 2:**
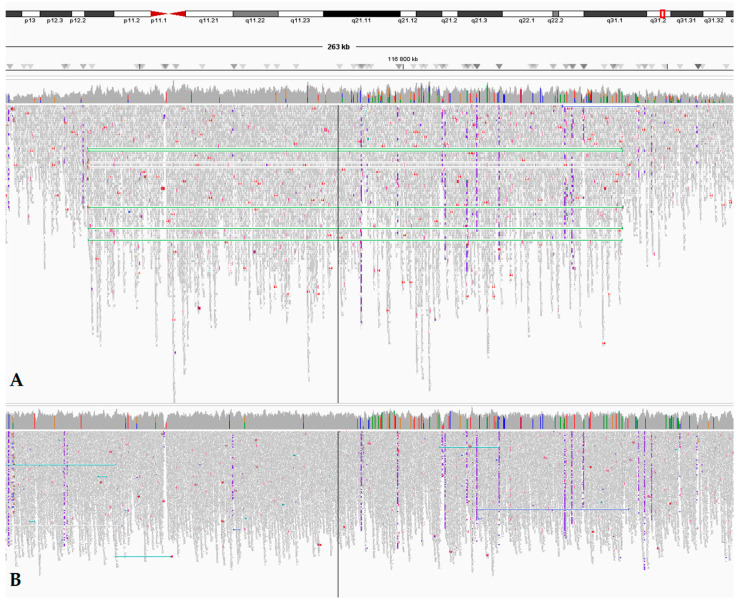
Germline *MET* exon 5-21 duplication detected by WGS in the probands. Legend: WGS analysis in Integrative Genomics Viewer (IGV) in a case with heterozygous duplication at locus 7q31.2 (chr7:116740252-116841718). Samples from patient #6 (**A**) and a healthy individual (**B**) are presented.

**Case #1**: Of the 175,467 identified variants different from the reference genome, no P/LP germline point genetic variants were identified, including *MET* missense mutations associated with hereditary/congenital cancer or renal pathology. Yet, we detected an extended heterozygous duplication of 101,467 kB (chr7:116740252-116841718; hg38) at the 7q31.2 locus, leading to an additional copy of the *MET*, including exons 5-21 (Figure 2). It should be noted that the duplicated exons encode most of the receptor domains. However, according to the ACMG criteria, at the present time, the identified duplication is classified as a VUS. Since the proband’s parents have passed away, it is not possible to obtain biomaterial from them for duplication validation. The patient’s elder brother was informed about the necessity of testing for this duplication.

**Figure 3:**
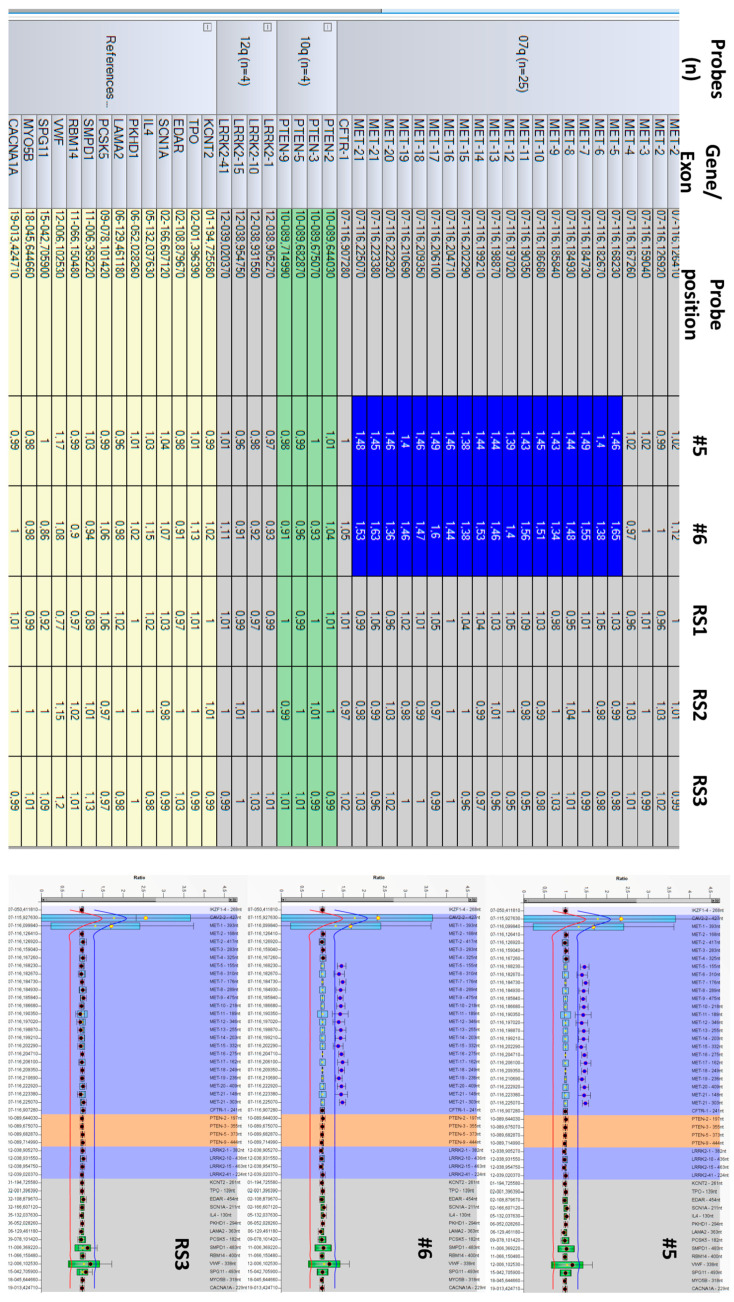
MLPA result (plots from Coffalyser software). Legend: #5 and #6—brothers from pedigree of Case #1; RS1-3—reference (normal) genomic DNA samples.

We have confirmed the *MET* duplication by an independent method, MLPA (Figure 3). The proband and his brother (#6 and #5 in the pedigree, respectively, Figure 1) harbor a heterozygous duplication (gain) of *MET* exons-5-21.

**Case #2:** Of the 171,141 identified variants different from the reference genome, no P/LP germline point genetic variants were identified, including *MET* mutations. However, we detected an extended heterozygous duplication of 101,467 bp (chr7:116740252-116841718) in the patient, like in Case #1. In addition, a secondary finding was detected in the patient—a germline variant in the *PALB2* gene (NM_024675.4): chr16:23635666dup (hg38), c.886dup (p.Met296AsnfsTer7) with a depth ×45 reads. We have classified this variant as likely pathogenic according to the ACMG criteria. The identified variant is included in the list of secondary findings of ACMG v3.2, according to which P/LP variants detected during exome or genomic sequencing in *PALB2* should be reported by the laboratory geneticist [15], and we have submitted this record.

## 4. Discussion

Molecular genetic diagnostics allowed us to identify a germline heterozygous duplication of exons 5-21 in the *MET* gene of 101,467 bp in the probands. This is attributed to a VUS according the ACMG guidelines at the present time (NC_000007.14: g.116740252_116841718dup). This germline *MET* duplication in patients with suspected HPRC is described by us for the first time according to open data. Analysis using the UCSC browser (https://genome.ucsc.edu/, accessed on 3 April 2025) revealed that similar duplications of the *MET* gene region including exons 5-21 have been described: the 98,697 bp duplication (chr7:116740619_116839316dup) was indicated in the DECIPHER database in patient #505381 as a result of clinical testing without specifying the phenotype and the 57,535 bp duplication (chr7:116740852-116798386dup) in a patient with kidney cancer without specifying the tumor type. The databases also contain information about the germline duplication chr7:116699075-117504373dup of 805,299 bp, two patients with a duplication of approximately 1.2 kb with exon 2 of *MET*, and two patients with a duplication of 181 bp including exon 6 of the *MET* gene. Notably, all of the cases mentioned above were diagnosed in patients with papillary renal carcinoma or RC without specifying the carcinoma type (Table 1).

Germline duplications were annotated as VUSs during clinical testing in accordance with the ACMG recommendations [10] in all the above-described rare cases of RC (Table 1), as well as in our Cases #1 and #2. At the same time, the following suggestion can be made based on Table 1: Large duplications of hundreds of thousands or millions of bp that span *MET* and several other genes on chromosome 7 are often classified as P/LP variants, leading to neurodevelopmental disorders and skeletal abnormalities and are characterized by severe congenital diseases as a whole. However, most duplications that affected only part of the *MET* gene or its exon were described in patients with suspected HPRC or unspecified RC.

It could be suggested that duplications of the SEMA/other domain or a major part of the MET receptor can lead to the constitutive homodimerization and activation of these, thereby promoting carcinogenesis. A similar mechanism of activation with a homodimerization of the tyrosine kinase receptor or its part as a result of mutation is known, in particular, in the FGFR family genes and the *RET* gene [16,17]. A precise pathogenicity analysis of germline duplications that include only the *MET* gene is still complicated, because *MET* is not yet included in the ACMG list of genes for reporting of CNV secondary findings obtained as a result of exome or genomic sequencing in routine diagnostic practice [18].

*MET* duplication is estimated to be a pathogenic alteration with a probability of 0.99% using the pTriplo algorithm, which is designed to determine the probability of triplosensitivity (i.e., duplication intolerance) from an analysis of large rare copy numbers in 950,278 individuals. pTriplo scores were generated by a machine learning model to predict the likelihood that complete duplication of each gene would be enriched in a cohort of individuals with diseases as compared to the general population. However, pTriplo can only predict the pathogenicity of a whole gene duplication, not a partial one [19].

Case #2 was found to have an LP variant in the *PALB2* gene. This gene is a homologous recombination repair (HRR) gene family, and germline P/LP variants in the *PALB2* are causes of a hereditary cancer syndrome. *PALB2* mutation carriers have an increased risk of breast cancer (men and women), prostate cancer, ovarian cancer, pancreatic cancer, and some other types of carcinomas [20]. *PALB2* germline mutations are highly penetrant alterations among HRR genes and require genetic counseling for carriers [21]. Patient #2 was informed about the risk of developing cancer and the advisability of testing for close relatives. However, we did not find information about *PALB2* mutations as a possible cause of multiple papillary RC. Given the published geno-phenotypic features of *PALB2* cancer syndromes [22,23], there is no reason to consider the identified variant as a cause of the disease in Case #2, but it is not possible to completely exclude such a situation at this stage of the study.

Trisomy of chromosome 7, amplification and/or somatic activating mutations, and the associated overexpression of *MET* are frequent driver events in the carcinogenesis of sporadic papillary renal cell carcinoma [6,24]. Somatic mutations that influence the copy number of individual *MET* exons in processed mRNA have been described in other tumor types. For example, mutations involving *MET* exon 14 occur in 4% of non-small-cell lung cancer. As a rule, they are splice site mutations leading to the loss of the exon 14 sequence in mRNA. This region encodes a sequence that plays an important role in the ubiquitin-dependent degradation of MET. Loss of the ubiquitination site promotes the accumulation of functionally active MET and carcinogenesis. A study of 168 cases of lung cancer with skipping of *MET* exon 14 has demonstrated that 40% of cases are resistant to inhibitors of the MET tyrosine kinase domain [25]. It should be noted that *MET* point mutations have been described not only in exons 15-21. Activating somatic missense mutations with a carcinogenic effect have also been described in the N-domain of MET in sporadic papillary renal carcinomas and non-small-cell lung cancer, although, unlike activating missense variants in the tyrosine kinase domain, they were ligand-dependent [26].

As mentioned earlier, amplifications, duplications, splice site mutations with loss of exon 14 in mRNA, and point mutations in the tyrosine kinase domain, but not duplications of the extracellular domains of MET, were observed in hereditary and sporadic neoplasms. A case with somatic duplication of the *MET* gene region containing exons 3-5 and encoding the SEMA domain in metastatic non-small-cell lung cancer was published in 2020. Functional studies confirmed the oncogenic character of this variant—the SEMA domain promotes the formation of the HGF binding site and the dimerization and activation of the receptor. Decreasing tumor size and a full response to crizotinib were observed in this case [27]. This case study shows that *MET* duplications encoding not only the intracellular but also the extracellular part of the receptor can be oncogenic and have predictive significance of I-II tiers according to AMP/ASCO/CAP recommendations.

The publication of rare clinical cases allows us to better understand the spectrum of molecular genetic alterations leading to HPRC [28,29]. If the pathogenicity of the identified *MET* duplication is confirmed in the future, such cases can be considered HPRC. The first step was the validation of the *MET* duplication in the patient’s brother with HPRC (Case #1), who was revealed to also harbor this germline variant. Genetic testing would be advisable for other living relatives who were not available for genetic counseling at the present time. Unfortunately, DNA samples of proband #1’s parents are not available for analysis of germline variants, and the oncological disease in the patient’s mother was diagnosed after 55 years, so it may be sporadic. However, if a segregation of *MET* duplication with HPRC is observed in the brothers (no. 3–6, Figure 1), then, considering the number of meioses, it will be possible to apply the supporting PP1 pathogenicity criterion according to the ACMG guideline and reclassify the *MET* duplication towards an LP variant. In this case, a recommendation will be given to test the proband’s children (no. 8–9, Figure 1) for this duplication and provide a dynamic observation of all mutation carriers to diagnose RC in time. Further, functional studies are necessary to clarify the pathogenicity of the duplication.

Currently, genetic testing for germline *MET* mutations is recommended for patients who meet at least one of the following criteria: (1) a family history of HPRC, (2) pathogenic *MET* germline variant in a close relative, (3) an individual history of multiple papillary renal carcinomas, (4) papillary renal cancer at the age of under 45 years. Since HPRC is a relatively indolent disease, it is possible to observe the patient and remove tumors during small part resections, as indicated in the National Comprehensive Cancer Network (NCCN) guidelines [30]. Targeted drugs for systemic therapy of HPRC are being developed. In particular, the MET and VEGFR2 inhibitor foretinib showed a significant response rate specifically in germline versus somatic *MET* mutations (50 vs. 5%). However, in this study, patients with germline mutations carried missense variants, while patients with somatic mutations were mostly characterized by *MET* CNVs [6,31]. Therefore, it cannot be excluded that a patient with a germline duplication will respond differently to MET inhibitor therapy than a patient with a germline missense mutation. Other tyrosine kinase inhibitors, crizotinib and savolitinib (especially in combination with the PD-L1 inhibitor durvalumab), demonstrate a pronounced response in MET-mutated tumors [32].

## 5. Conclusions

Thus, we have described, for the first time, a germline ~101.4 kb duplication including a large part of the *MET* gene with exons 5-21 in three patients with clinical features of HPRC. A conclusion about the pathogenicity and clinical significance of this duplication will be possible after providing functional studies and observations of rare families with similar *MET* aberrations. At the same time, we are of the opinion that timely reporting of rare germline findings obtained by NGS is important for understanding the possible genetic causes of HPRC, as well as for genetic CNV variant reclassification.

## Figures and Tables

**Table 1:** Germline duplications affecting the *MET* gene and their clinical–genetic correlations.

Coordinates, GRCh38 (chr7)	Duplication Size, bp	Duplicated *MET* Exon(s)	Phenotype	Sample Reference
97419852–158923762	61,503,911	All exons and flanking genes	Developmental disabilities, congenital anomalies	147547 ^1^
115459015–159325817	43,866,803	All exons and flanking genes	Not reported	152912 ^1^
115059311–132281500	17,222,190	All exons and flanking genes	Short stature, autism, gait imbalance, delayed gross motor development	322280 ^2^
114578028–125438062	10,860,035	All exons and flanking genes	Autism, intellectual disability, bulbous nose, low-hanging columella, thin upper lip vermilion	2362 ^2^
114105646–116927595	2,821,950	All exons and flanking genes	Hypotonia, abnormal pinna morphology, high and narrow palate, wide nasal bridge, feeding difficulties, adducted thumb, single transverse palmar crease, aplasia hypoplasia of the lungs, apnea	439826 ^2^
115015244–116710591	1,695,348	1–2 and 5′-flanking genes	Motor delay, mild intellectual disability	509153 ^2^
115042242–116687714	1,645,473	Promoter and 5′-flanking genes	Developmental disabilities, congenital anomalies	59715 ^1^
116699075–117504373 *	805,299	2–21 and 3′-flanking genes	Papillary RC	583430 ^1^
116756194–117318372	562,179	6–21 and 3′-flanking genes	Hypotonia, seizure disorder, autism with high cognitive abilities, mild global developmental delay, hypertelorism, prominent forehead	281389 ^2^
116740252–116841718 *	101,467	5–21	Papillary RC	Cases #1 and #2
116740619–116839316	98,698	5–21	Not reported	505381 ^2^
116740852–116798386 *	57,535	5–21	RC (type not defined)	252900 ^1^
116699075–116700294 *	1220	2	Papillary RC	583929 ^1^
116699084–116700284 *	1201	2	RC (type not defined)	220130 ^1^
116755345–116755525 *	181	6	Papillary RC, RC (type not defined)	643144 ^1^ (2 samples)

^1^ Variation ID in the ClinVar (http://www.ncbi.nlm.nih.gov/clinvar/, accessed on 3 April 2025); ^2^ patient’s ID in the DECIPHER CNV browser (https://www.deciphergenomics.org/, accessed on 3 April 2025), * duplication in a patient with RC; duplications are listed in decreasing order of size.

## Data Availability

Sanger sequencing chromatograms of the *MET* and *FH* genes and the NGS data files can be provided upon reasonable request.

## Abbreviations

The following abbreviations are used in this manuscript:

HPRC	Hereditary Papillary Renal Carcinoma
ACMG	American College of Medical Genetics and Genomics
VUS	Variant of Uncertain Significance
RC	Renal Cancer
WGS	Whole-Genome Sequencing
WHO	World Health Organization
ISUP	International Society of Urological Pathology
P/LP	Pathogenic/Likely Pathogenic
MRI	Magnetic Resonance Imaging
USA	The United States of America
PCR	Polymerase Chain Reaction
MLPA	Multiplex Ligation-dependent Probe Amplification
DNA	DeoxyriboNucleic Acid
NGS	Next Generation Sequencing
HGVS	Human Genome Variation Society
CNV	Copy Number Variation
OMIM	Online Mendelian Inheritance in Man
FIP	Federal Institute of Industrial Property (Russia)
IGV	Integrative Genomics Viewer
HRR	Homologous Recombination Repair
AMP	Association for Molecular Pathology
ASCO	American Society of Clinical Oncology
CAP	College of American Pathologists
NCCN	The National Comprehensive Cancer Network
CGC	Cancer Genomics Consortium
VICC	Variant Interpretation for Cancer Consortium
